# An Improved Method for Including Upper Size Range Plasmids in Metamobilomes

**DOI:** 10.1371/journal.pone.0104405

**Published:** 2014-08-12

**Authors:** Anders Norman, Leise Riber, Wenting Luo, Li Li Li, Lars Hestbjerg Hansen, Søren Johannes Sørensen

**Affiliations:** 1 Section of Microbiology, Department of Biology, University of Copenhagen, Copenhagen, Denmark; 2 Department of Earth and Planetary Science, University of California, Berkeley, California, United States of America; 3 Department of Environmental Science, Aarhus University, Roskilde, Denmark; University of Manchester, United Kingdom

## Abstract

Two recently developed isolation methods have shown promise when recovering pure community plasmid DNA (metamobilomes/plasmidomes), which is useful in conducting culture-independent investigations into plasmid ecology. However, both methods employ multiple displacement amplification (MDA) to ensure suitable quantities of plasmid DNA for high-throughput sequencing. This study demonstrates that MDA greatly favors smaller circular DNA elements (<10 Kbp), which, in turn, leads to stark underrepresentation of upper size range plasmids (>10 Kbp). Throughout the study, we used two model plasmids, a 4.4 Kbp cloning vector (pBR322), and a 56 Kbp conjugative plasmid (pKJK10), to represent lower- and upper plasmid size ranges, respectively. Subjecting a mixture of these plasmids to the overall isolation protocol revealed a 34-fold over-amplification of pBR322 after MDA. To address this bias, we propose the addition of an electroelution step that separates different plasmid size ranges prior to MDA in order to reduce size-dependent competition during incubation. Subsequent analyses of metamobilome data from wastewater spiked with the model plasmids showed *in silica* recovery of pKJK10 to be very poor with the established method and a 1,300-fold overrepresentation of pBR322. Conversely, complete recovery of pKJK10 was enabled with the new modified protocol although considerable care must be taken during electroelution to minimize cross-contamination between samples. For further validation, non-spiked wastewater metamobilomes were mapped to more than 2,500 known plasmid genomes. This displayed an overall recovery of plasmids well into the upper size range (median size: 30 kilobases) with the modified protocol. Analysis of *de novo* assembled metamobilome data also suggested distinctly better recovery of larger plasmids, as gene functions associated with these plasmids, such as conjugation, was exclusively encoded in the data output generated through the modified protocol. Thus, with the suggested modification, access to a large uncharacterized pool of accessory elements that reside on medium-to-large plasmids has been improved.

## Introduction

Plasmids are extrachromosomal mobile genetic elements (MGEs) that constitute the foundation of communal gene pools within select microbial settings [1]. A classical illustration of their potency is the process by which persistent selective pressures, such as the widespread overuse of antimicrobial agents, can lead to accretion of genes onto plasmids. These, in turn, will disseminate rapidly throughout environments in which their genetic load confers the appropriate selective advantage. Thus, as mobile gene-carriers, they represent a vast and highly dynamic resource that enables poorly adapted bacteria to tap into a much larger reservoir of genes to acquire new functions [2], [3]. So far, only limited information about plasmid ecology, or the specific roles that plasmids actively play within microbial systems *in situ* has been made available. Yet, basic knowledge about how they operate as early responders to local environmental perturbations, or as agents of lateral gene transfer, is key to understanding the natural evolution, accretion and propagation of microbial genes [4].

Large conjugative plasmids have an intrinsic ability to transport multiple independently operating gene cassettes, but also carry with them a tightly regulated collection of conserved vital plasmid-selfish genes, which makes them particularly good candidates for further study [1], [5]. Early attempts at establishing their ecology have been dominated by PCR amplification of marker genes or loci, such as replicases, relaxases and origins of replication [6], [7]. Others have tried to survey genetic loads carried by these plasmids in specific settings (e.g. soil or wastewater), either through exogenous isolation into culturable conjugation recipients [8] or by sequencing of contrived “plasmid communities” comprised of pooled plasmid purifications from resistant plate colonies [9].

Recent efforts have seen a concerted move toward more metagenomic approaches, as was previously seen with virus metagenomes (hence, “viromes”) [10]. However, such “plasmidomes” [11] (we have previously used the term metamobilome [12], [13]) are considerably less tractable, as plasmids almost exclusively inhabit the cytoplasm of their host, precluding enrichment through simple filtration of cell suspensions. Metamobilome studies must therefore take steps to minimize, if not eliminate, the contribution from the more abundant chromosome fragments (gDNA). This is a crucial step, as only a negligible fraction of the total community will consist of plasmids, and subsequent data analyses are rendered infeasible by an overwhelming presence of non-plasmid data [14].

The most direct way of achieving pure plasmid DNA (pDNA) for metagenomic analyses is to employ one of the many standard purification techniques designed for isolating extrachromosomal circular DNA directly in environmental bulk lysates. Sentchilo *et al.*, for example, have used classic CsCl density gradient centrifugation, combined with Sanger and 454-sequencing, to characterize a wastewater metamobilome, from which they were able to derive several large plasmids [15]. Although very high pDNA purity can be achieved through this technique, CsCl gradient centrifugation is a cumbersome and delicate procedure, which relies heavily on expensive equipment (an ultracentrifuge) and fairly toxic chemicals.

A different and arguably more tractable approach is to purge gDNA from samples by subjecting them to a cocktail of exodeoxyribonucleases, such as the ATP-dependent DNase from *Micrococcus luteus* combined with *Escherichia coli* exonuclease I [16]. This will degrade linear fragments (gDNA), leaving only circular (both closed and open/nicked) pDNA under optimal circumstances, when applied to either bulk lysates or plasmid purifications. Exonuclease reactions therefore have to be monitored closely for gDNA (using 16S rRNA gene primers), which usually remains detectable for up to 48 hours after the beginning of the reaction [17]. Subsequently, the often scarce quantities of remaining pDNA must to be raised to levels suitable for high-throughput sequencing. This can be achieved through multiple strand displacement amplification (MDA), such as the one carried out by the rolling-circle amplifying (RCA) Φ29 DNA polymerase [18]. A few variations on these few basic steps have previously been carried out in wastewater [12], [14], bovine rumen [17], [19] and rat cecum samples [13]. However, these studies have shown an apparent limitation in the recovery of plasmids and other circular DNA that exceed 10 Kbp. One possible explanation for this phenomenon is that RCA naturally favors smaller circular elements over larger ones (see discussion), which in turn could lead to over-representation of predominantly smaller (1–10 Kbp) plasmids to the exclusion of larger plasmids.

In the current study, we draw attention to the potentially strong bias, contributed by MDA. We propose the addition of an electroelution step to the protocol, demonstrate how this enables more efficient capture of upper size range plasmids (>10 Kbp) and how this affects downstream data output.

## Materials and Methods

### Ethics Statement

No specific permissions were required for the described locations/activities. No specific permits were required for the described field studies because these did not involve sample collection affecting endangered or protected species.

### Bacterial strains, plasmids and growth conditions

Model plasmids pBR322 (4.4 Kbp) [20], and the Tet^R^-Kan^R^-Str^R^-*gfp*-tagged variant of IncP-plasmid pKJK5, pKJK10 (56 Kbp) [21], were propagated in *E. coli* CSH26 [K-12;F^-^, *ara*, Δ(*lac*-*pro*), *thi*] growing in Luria-Bertani (LB) medium at 37°C, supplemented with either 100 µg/mL ampicillin and 10 µg/mL tetracycline (pBR322) or 50 µg/mL kanamycin, 100 µg/mL streptomycin and 10 µg/mL tetracycline (pKJK10). Plasmid DNA was purified from overnight cultures using the QIAprep Spin Miniprep kit (Qiagen, Valencia, CA) for pBR322 and the Plasmid Mini AX kit (A & A Biotechnmology, Gdynia, Poland) for pKJK10.

### Plasmid DNA amplification assay

Exonuclease digestion reactions of pBR322, pKJK10 and the mixture of the two plasmids were prepared as described below, with all plasmids in concentration of ∼0.2 ng DNA/µL and run for 48 hours. Digestion products were washed twice with Sigma water and concentrated to 20 µL, using Amicon Ultra-0.5 mL Centrifugal Filters (Merck Millipore, Billerica, MA). DNA concentrations were measured on the Qubit Fluorometer (Life technologies, Carlsbad, CA). Multiple displacement amplification reactions were prepared as described below for up to 9 hours (see results section), with 1 µL of the digested plasmid DNA as template. Each reaction was carried out in triplicate.

### Electroelution recovery assay

Following electrophoresis on 1% agarose, the larger of the two bands, corresponding to pKJK10, underwent electroelution as described below. This procedure was reproduced a total of six times. The molecular integrity of the electroeluted pKJK10 was visually inspected on agarose gels to confirm the absence of low molecular weight bands (corresponding to pBR322), or smears (presence of sheared DNA). Recovery of pKJK10 and pBR322 was estimated by comparing copy numbers (estimated with qPCR as decribed below) before and after electroelution.

### Wastewater metamobilomes

Wastewater was collected from the primary sedimentation tank of a Danish sewage treatment plant that services 8–12 million m^3^ of wastewater from approximately 130.000 individuals annually (Lundtofte, Lyngby Denmark; 55° 48′ 8″ N, 12° 32′ 23″ E). Samples of 20 L were filtered (125 mm filters, Frisenette ApS, Knebel, Denmark), and cells from the filtrate were recovered by centrifugation (8000 g, 10 minutes) and washed in phosphate buffered saline solution (PBS). Total plasmid DNA isolations were done using the Plasmid Mini AX kit (A&A Biotechnology, Gdynia, Poland) on approximately 5 mg of wastewater pellet. The standard metamobilome (S-library) was generated as previously described [12], [13]. For the upper size range metamobilome (L-library), DNA fragments of the desired size (>10 Kbp) were recovered by electroelution as described below. Spiked libraries Ss and Ls were prepared as before, but from pellets where 2×100 µL of overnight culture of *E. coli* CSH26 hosting pBR322 and pKJK10, respectively, had been added beforehand.

### Modified mobilome isolation protocol with electroelution step

Following gel electrophoresis of purified plasmid DNA on 1% agarose, sections in the desired size-range of the gel (the area between the 23 Kbp marker band and the gel well) were excised from the appropriate lanes and placed in dialysis bags (Spectra/Por dialysis membrane, MWCO = 8000 Daltons, Spectrum Laboratories Incorporated, Rancho Dominguez, CA) containing TAE buffer [40 mM Tris, 40 mM acetate, 1 mM EDTA, pH 8.2]. Electroelution ran for 2 hours at 110 V with the electrophoresis chamber placed on ice. The current was briefly (20–30 s) reversed to release any DNA attached to the dialysis membrane prior to ethanol precipitation. Sheared gDNA and linearized pDNA was removed with Plasmid-Safe ATP-Dependent DNase (Epicentre, Madison, WI) in 50 µL reactions. The progression of exonuclease digestion was monitored by measuring the amount of remaining gDNA every 12 hours; specifically, this was done by extrapolating the number of genome copies from 16S rRNA gene-abundance as determined by qPCR (see below). Following 48 hours of exonuclease treatment, digestion reactions were indistinguishable from a non-template control. Reactions were stopped by heating to 70°C for 30 min.

The remaining pDNA was precipitated with isopropanol and amplified using the REPLI-g Mini kit (Qiagen, Valencia, CA) incorporating the modifications outlined by Pan *et al.* [22] which minimizes the generation of template independent products. Briefly, 1 µL pDNA was added to 1 µL freshly prepared buffer A [400 mM KOH, 10 mM EDTA] and placed on the bottom of a precooled PCR tube, which was then incubated for 3 minutes at room temperature. Following denaturation, the tube was transferred to ice and 1 µL neutralization buffer N [200 mM HCl, 300 mM Tris·HCl, pH (7.5)] was added, immediately followed by 5 µL of 1.2 M sterile-filtered Trehalose. Then, 32 µL of a Master Mix [1.2 M 27.0 µL Trehalose, 12.5 µL 4X REPLI-g Mini Reaction buffer and 0.5 µL REPLI-g DNA polymerase] was mixed with the 8 µL of denatured DNA solution. MDA reactions ran for 7 hours, unless otherwise stated, at 30°C, after which the REPLI-g DNA polymerase was heat-inactivated for 5 minutes at 65°C. Aliquots were stored at -20°C or at room temperature for up to one week.

### Quantitative PCR (qPCR)

Abundance of plasmids pBR322 and pKJK10 was monitored with qPCR using primer-sets BR1/2 (5′-GGTTATTGTCTCATGAGCGG & 5′-TTAGATTTCATACACGGTGCC) and GFP1/2 (5′-TCGGTTATGGTGTTCAATGC & 5′-GACTTCAGCACGTGTCTTGTAG), which targets the pBR322 multiple cloning-sites region and the pKJK10 green fluorescent protein *gfp*-gene, respectively (both of which are highly unlikely to occur naturally). Twenty microliter reactions were run on the Mx3000P qPCR system (Stratagene, Santa Clara, CA) using the 1× Brilliant SYBR Green Master Mix with 0.5 µM of each primer and 1 µL of template DNA in a total volume of 20 µL. The following program was run for each qPCR reaction: Initial denaturing at 95°C for 10 min, 40 cycles of 95°C for 30 s, 60°C for 1 min and 72°C for 1 min, followed by one cycle of 95°C for 1 min, 55°C for 30 s and 95°C for 30 s.

For monitoring the presence of sheared chromosomal DNA, a standard was first made from *E. coli* K-12 MG1655 gDNA; extracted using the Genomic Mini AX kit (A&A Biotechnology, Gdinya, Poland) and assayed on an Invitrogen's Qubit fluorometer. Universal 16S rRNA-gene specific primers Eub338/518 [23] were used to monitor gDNA-levels during qPCR, as described above. Threshold values, standard curves and amplification efficiencies were calculated using MxPro qPCR software version 4.10.

### Illumina high-throughput sequencing

Template DNA was fragmented using a bioruptor (Diagenode, Liège, Belgium), and products of 200–500 bp were recovered using the MinElute PCR Purification kit (Qiagen, Valencia, CA). This was followed by end repair, phosphorylation, dA-tailing and adaptor ligation using the respective NEBNext-modules (New England Biolabs, Ipswich, MA) to prepare samples for Illumina sequencing. Libraries were then tagged using short indexing amplification primers as previously described [24] and sequenced as multiplexed single-read libraries for 100 cycles (including a 6 cycle index read) on a single lane of an Illumina HiSeq2000 flowcell, according to the manufacturer's protocol.

### Sequence data analysis

All general sequence manipulation and analysis was carried out using the Biopieces software suite (www.biopieces.org). Low quality HiSeq Reads (<Q20) were filtered, and reads shorter than 20 bp were discarded. Since sequencing only provided short single reads, metagenomic assemblies were done using meta-idba with a kmer-range of 19–79 [25], which performed better than velvet [26] on all samples. Putative coding sequences were identified with prodigal [27]. Read-mapping to genomes and the assembled contigs was done with the Burrows-Wheeler aligner bwa [28]. All predicted protein sequences were screened for conserved motifs using hmmscan (http://hmmer.janelia.org/) against the Pfam-A and Pfam-B databases (http://pfam.sanger.ac.uk/) using the –cut_ga option (gathering-threshold cutoff). The genome, plasmid and phage databases were downloaded from the NCBI Reference Sequence Database (RefSeq) NCBI online repository on October 25^th^, 2011 (ftp://ftp.ncbi.nih.gov/refseq/release/). The pBR322 nucleotide sequence, which is not present in RefSeq, was downloaded directly from NCBI GenBank (accession no.: J01749). In lieu of a full pKJK10 sequence, pKJK5, which pKJK10 is directly derived from [21], and the *gfp*mut3*-variant of the *Aequorea victoria* gene *gfp* were used for mapping. The *gfp*mut3* nucleotide sequence was retrieved from cloning vector pBR939b (accession no: JQ394798). To qualify for “partial coverage” a genome sequence was required to meet the following criteria in at least one out of four metamobilome libraries: (1) a minimum of 1,500 bases covered by one or more reads, (2) at least 10% genome coverage, and (3) average coverage over all covered base positions exceeding 1×. This was to minimize the number of plasmids that only recruited substantial numbers of reads due to presence of common repeat elements, such as IS sequences, or plasmids with extremely poor coverage. Any sequence meeting the previous criteria, but with at least 85% genome coverage, was categorized as having “near complete to complete coverage”.

LS-skew values, used to determine the relative contributions of sequencing reads mapped from the L- and S-libraries, respectively, were calculated according to 
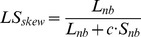
where *nb* indicates number of bases mapped between the first and last bases of a contig or gene, and the constant *c* (0.95) was used to correct for a small difference in sizes between the L-library and the S-library. Thus, an LS_skew_ of 0 indicates all mapped reads originated from the S-library, while an LS_skew_ of 1 means all reads originated from the L-library. In all instances, sequences were considered to be exclusive to the S-library when LS-skew<0.05 and exclusive to the L-library when LS-skew>0.95. Otherwise sequences were seen as stemming from both libraries.

## Results

### Assessment of enrichment bias in mobilome isolations based on exonuclease treatment and MDA, and recovery of large plasmids with electroelution

**Figure 1:**
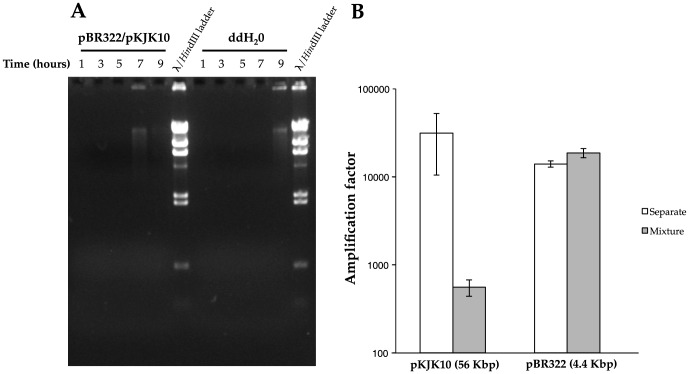
Optimization (a) of multiple displacement amplification (MDA) incubation time, and (b) measured fold-amplification of plasmids pBR322 (4.4 Kbp) or pKJK10 (56 Kbp), following 7 hours of MDA. The two plasmids were mixed in equal concentration (∼0.2 ng/µL) and subjected to plasmid-safe exonuclease digestion for 48 hours, followed by MDA for 1, 3, 5, 7 or 9 hours. MDA products were visualized on 1% agarose to estimate the longest possible MDA incubation time (panel a, left lanes) that did not lead to generation of template-independent products (panel a, right lanes). Quantitative PCR (qPCR) was used to quantify copies of pBR322 and pKJK10 before and after 7 hours of MDA, which was performed on plasmids in separate reactions as well in mixture. Error bars represent standard deviations of three independent replicates.

The utilization of MDA for amplifying pDNA, potentially introduces a source of bias toward small circular elements due to this technique being based on the Φ29 (RCA-type) DNA polymerase. Therefore, we first aimed to evaluate the enrichment bias in favor of small plasmids when using the established mobilome isolation method [12], [13]. For this, plasmid purifications of pBR322, pKJK10, and a mixture of the two, respectively, were subjected to exonuclease digestion to remove gDNA at a starting concentration of 0.2 ng/µL. Following a 60% reduction in volume, the DNA concentrations of all three samples were 0.14 (± 0.03) ng/µL. Thus, 62–80% DNA had been removed from all reactions by exonuclease, with little to no discernible difference between the three different reactions. The three reactions and the non-template control were subsequently subjected to MDA for 1, 3, 5, 7 and 9 hours. Gel-electrophoresis was used to estimate the optimal amplification time by monitoring for appearance of a band in the non-template control compared to the mixed plasmid reaction, indicating background DNA amplification and/or generation of template-independent products. This occurred after 9 hours of incubation (Figure 1a), and 7-hour reactions were therefore selected for further analyses. The relative increases in plasmid copies following MDA were determined with qPCR using pBR322 and pKJK10-specific primers (see methods). Both plasmids were amplified in comparable numbers when amplified separately, but pKJK10 saw a more than 50-fold reduction in amplification in the pBR322/pKJK10-reaction, compared to the separate pKJK10-reaction (Figure 1b). Conversely, pBR322 was relatively unaffected by the presence of pKJK10 and was amplified 34 (±4) times more than pKJK10 in the mixed plasmid reaction (Figure 1b). Thus, the plasmid mixture that started with the two plasmids in equal DNA concentrations, ended up consisting of 97% pBR322 and 3% pKJK10.

To evaluate the recovery of upper size range (>10 Kbp) plasmids with the suggested new electroelution step in the mobilome isolation protocol, purifications of plasmids pBR322 and pKJK10 were mixed in equal concentrations as before and subjected to the electroelution step. Average recoveries were measured at 12.4% (± 6.8) for pKJK10 and 0.07% (± 0.001) for pBR322. Thus, the electroeluted fraction of the plasmid mixture consisted of 99.4% pKJK10 on average.

### Estimating size ranges of plasmids recovered from wastewater metamobilomes with the established- and modified protocol, using read recruitment to known plasmid sequences

**Figure 2:**
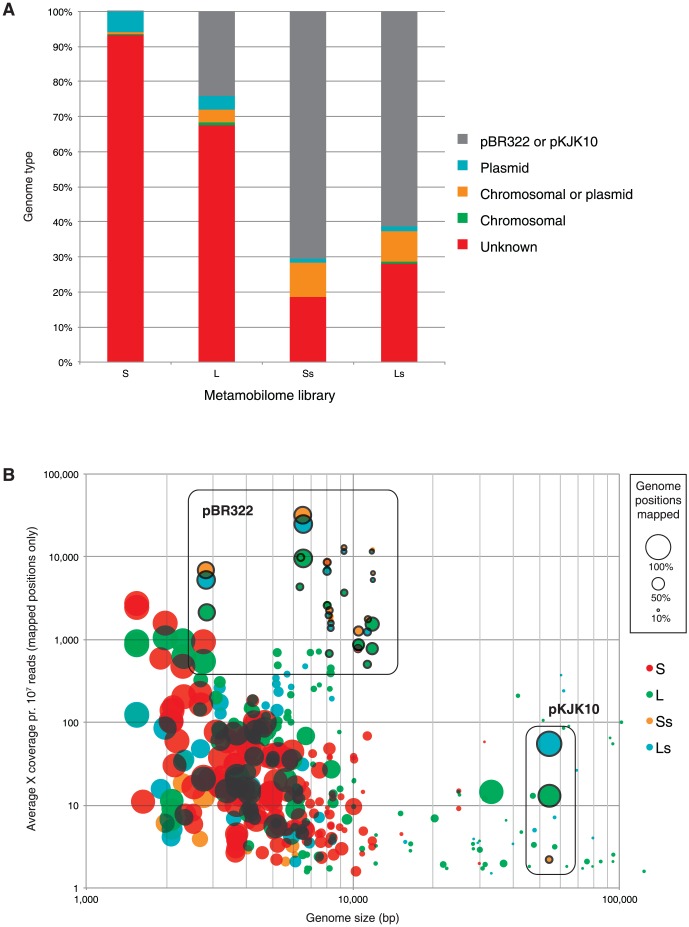
Recruitment of Illumina sequencing reads from wastewater metamobilomes generated using an established isolation protocol (S) and the modified protocol (this study) using electroelution (L), along with two corresponding libraries from wastewater spiked with model plasmids pBR322 and pKJK10 (Ss & Ls). The bar graph (a) shows the composition of read-hits to either model plasmid, or sequences within the NCBI RefSeq databases of fully sequenced plasmids or chromosomes. Reads hitting neither of the two model plasmids, or any sequence within RefSeq are labeled as unknown. The bubble diagram (b) shows an overview of the coverage- and size-distribution of plasmid genomes in the RefSeq database with significant read-recruitment from one or more metamobilomes. Plasmid sequences with strong homology to more than 1 Kbp of pBR322 or pKJK10 (which are both absent from the RefSeq database) are outlined in black.

In order to evaluate sequencing output from the two different isolation methods, a total of four metamobilome libraries were constructed from a wastewater bacterial community: A standard metamobilome (S-library), prepared as previously described [12], a metamobilome implementing the proposed electroelution step to target upper size range plasmids (L-library) and two similar libraries (Ss- and Ls-library) prepared as before, but from wastewater pellets spiked with a 1∶1 mixture of overnight culture *E. coli* harboring pBR322 and pKJK10, respectively. A total of 2.4 Gbp nucleotide data was produced from Illumina sequencing (Table 1). To estimate the size distribution of plasmids recovered with the two different isolation methods, reads from each of the four libraries were mapped against all completed microbial chromosome, plasmid and phage sequences present in the NCBI Refseq database (Figure 2). The proportion of reads that remained unmapped were 93% in the S-library, 68% in the L-library, and 18% and 28% in the corresponding spiked libraries, respectively. Samples contained between 0.05% and 0.83% reads that mapped exclusively to chromosomes, and between 0.42% and 10% that mapped to both plasmids and chromosomes. Between 6% and 72% reads mapped exclusively to plasmids (Figure 2a). No sample was found to map more than 0.1% of its reads to known phages (Table S1). The difference between the non-spiked and spiked libraries was primarily caused by massive recruitment of reads to a number of plasmids in the RefSeq database that contain stretches of sequence identical to the pBR322 or pKJK10 genomes (neither of these two plasmids are themselves included in RefSeq on account of being artificially created).

**Table 1:** Summary of reads generated by Illumina HiSeq2000 sequencing of four different wastewater metamobilomes.

Library	# of reads	Mean read length (bp)	Bases (Mbp)
S	8,403,018	78	657
Ss (spiked)	5,907,001	77	453
L	8,180,991	77	626
Ls (spiked)	8,544,867	76	652
Total	31,035,877	77	2,388

Two of these were spiked with plasmids pBR322 and pKJK10.

To better evaluate the recovery of the two model plasmids, another read mapping was performed, where only model plasmid sequences were present (Table 2). Reads that mapped to the model plasmids, constituted 70% and 61% of the Ss- and Ls-libraries, respectively (Figure 2a). The Ls-library provided complete mapping of pKJK10, with more than 99% of the sequence covered to a mapping depth greater than 50× (per 10 million reads), while the Ss-library only provided sparse (37%) coverage to a much more limited depth (3.3×). In both cases, pKJK10 was overwhelmingly outnumbered by reads mapping to pBR322, which was completely covered to mapping depths in excess of 100,000× in both libraries. However, the Ls-library, with its markedly improved recovery of pKJK10, only saw a 102-fold difference between pBR322- and pKJK10-mapped reads, compared to a 1,263-fold difference in the Ss-library (Table S1).

**Table 2:** Coverages^a^ of model plasmids^b^ from spiked and non-spiked wastewater metamobilomes.

Library	pBR322	pKJK5	*gfp*mut3*-gene
S	150 (99.9%)	1.4 (2.7%)	0.0 (0%)
L	45,000 (100%)	21 (96%)	12 (100%)
Ss	140,000 (100%)	3.3 (37%)	1.0 (100%)
Ls	120,000 (100%)	78 (99.5%)	53 (100%)

Numbers in parenthesis indicate the percentage of base positions covered by sequencing reads.

a Defined as X-coverage pr. 10 million Illumina reads.

b Due to a lack of a complete pKJK10-sequence, pKJK5, which covers 97% of the pKJK10-sequence, was used instead, along with the 755 bp *gfp*mut3*-gene, which is embedded in the Tn10-transposon inserted on pKJK10.

The L-library also provided considerable mapping of both model plasmids (see discussion). In fact, 24% of reads from the L-library mapped to pBR322, giving it complete coverage, while 0.16% of reads mapped to pKJK10, giving near-complete coverage (Table 2). Reads that mapped to plasmids in RefSeq, but neither of the model plasmids, constituted 6% and 4%, respectively, in the two non-spiked libraries, and about 1% in the two spiked libraries (Table S1). As the spiked wastewater sample had been run on the same gel as the non-spiked sample, this raised the concern that the elecroelution procedure was susceptible to cross-contamination. We therefore ran the 1∶1 mixture of pBR322 and pKJK10 on a separate gel and performed electroelution of gel-slices immediately next to, or two or three lanes away (in both directions) from the sample, respectively. Each gel-slice was electroeluted as above and tested for presence of pBR322 and pKJK10 using the two primer-sets mentioned in the methods section. Each gel-slice tested positive for both model plasmids. In fact it was possible to get a gel slice cut from an empty gel, run in a separate electrophoresis chamber, to test positive by performing the electroelution step in the same electrophoresis chamber as the other gel-slices (data not shown). All electro-eluates, which tested positive for both model plasmids, also tested positive following MDA.

Out of more than 2,500 plasmid genomes present in the RefSeq database, 148 qualified for partial coverage (see methods), and 61 qualified for near-complete to complete coverage (). Out of these plasmid-hitting reads, only 13% hit plasmids larger than 10 Kbp in the S-library, while 44% did the same in the L-library (following removal of model plasmid reads). A graphical overview of the read recruitment to RefSeq plasmids is shown in Figure 2b. Based on LS-skew values (see methods) assigned to each plasmid sequence with partial or more coverage, the median sizes of plasmids found in the S-, the L-, or both libraries, were calculated (Table 3). This showed the S-library to have a median plasmid size of 4.3 Kbp and the L-library to have a median plasmid-size of 30 Kbp. Approximately half of the RefSeq plasmids with partial or more coverage (92) were found to be present in both libraries and had a median length of 5.2 Kbp. The near-complete or complete plasmids originated from hosts primarily within the phyla Proteobacteria, Actinobacteria and Bacteroides, and ranged from 1.5–54.4 Kbp in size and 2.5×–2,100× coverage (Table S1). Only 2 plasmids from the L-library qualified for near-completeness: the IncP1-plasmid pKJK5 and the *Bacteroides* plasmid p5482. However, these plasmids were both larger than 10 Kbp (54 and 33 Kbp, respectively).

**Table 3:** Median lengths (Kbp), with lower- (Q1) and upper (Q3) quartiles, of RefSeq plasmids mapped by Illumina sequencing reads from metamobilomes S (standard isolation protocol) and L (modified electroelution protocol).

	RefSeq plasmids with partial- to complete coverage				RefSeq plasmids with near-complete to complete coverage			
Library	*N*	Median (Kbp)	Q1	Q3	*N*	Median (Kbp)	Q1	Q3
All	209	6.1	4.1	10.9	61	3.7	2.7	4.3
Only S	56	4.3	3.5	5.6	22	3.6	2.6	4.2
Both S & L	92	5.2	3.7	7.1	37	3.6	2.7	4.2
Only L	61	29.9	9.5	60.1	2	43.7	38.4	49.0

### *De novo* assembly and gene content analyses of metamobilomes generated with established and modified isolation protocols

To evaluate how the electroelution step affects downstream data output, sequenced reads from the two non-spiked libraries underwent *de novo* assembly (Table 4). Even though the two libraries were very similar in size, and both had approximately two thirds of their reads assembled, the S-Library on its own produced more than twice as much contig data as the L-library. The L-library, however, showed a slightly higher average contig length over the S-library, and had 9 sequences exceeding 5 Kbp in length. Additionally, a mixed assembly, in which reads from both libraries were combined, was performed in order to evaluate overlaps in (plasmid) genome content. In this assembly, slightly more reads were assembled than in the former separate assemblies, with each library contributing roughly the same number of reads. By assigning LS-skew values (see methods) to each contig in the mixed assembly, three fractions out of the resulting 7.1 Mbp of contig data could be distinguished: Those that were assembled almost exclusively from reads from the S-library (3.3 Mbp); those that were assembled with significant contributions from both libraries (2.8 Mbp); and those that were assembled almost exclusively by reads from the L-library (1.0 Mbp; data not shown). Again, contigs exclusive to the L-library were slightly longer on average, and had the same number of sequences (9) exceeding 5 Kbp in length. Nineteen contigs out of 1,048, ranging from 1.6 Kbp to 3.9 Kbp, had overlapping ends (14–78 bp overlaps of 100% nucleotide identity, identified using BLAST analysis; data not shown), suggesting that they were likely closed circular sequences. However, without additional paired-end information or verification of overlaps with PCR, confirming circularity of these sequences would be difficult [13], and was therefore not pursued any further. Their distribution among the three fractions (S-exclusive; both; or L-exclusive) was 10∶7∶2, with the largest contig being exclusive to the L-library.

**Table 4:** Summary of MetaIDBA assemblies made with Illumina reads from wastewater metamobilomes.

Library	Reads assembled (%)	Length of assembly (Mbp)	Longest contig (Kbp)	N50^a^ (bp)	No. of contigs >1 Kbp	Length of >1 Kbp contigs (Mbp)
S	66.1	5.44	6.6	459	817	1.25
L	67.8	2.51	7.4	567	476	0.86
Mixed assembly (S + L)	69.4	7.12	7.4	438	1,048	1.71

a N50 is defined as the length at which 50% of all bases in the sequences are in a sequence shorter than N.

To determine functional differences in gene content between the two libraries, genes were predicted on all contigs from the mixed assembly. Only putative genes containing both valid start and stop-codons were retained, which resulted in 5,343 coding sequences, ranging from 30 to 1053 aa. Out of these, 3,627 had sufficient coverage (>1×) for further analysis. Similarly to the contig analysis, genes were assigned individual LS-skew values so the contributions from the two libraries could be determined. Unsurprisingly, a similar proportion of the putative genes (69%) were exclusive to either of the two libraries, and the S-Library distribution of genes (1975∶1122∶530) was in accordance with the observed combined lengths of contigs from each fraction of the assembly.

**Figure 3:**
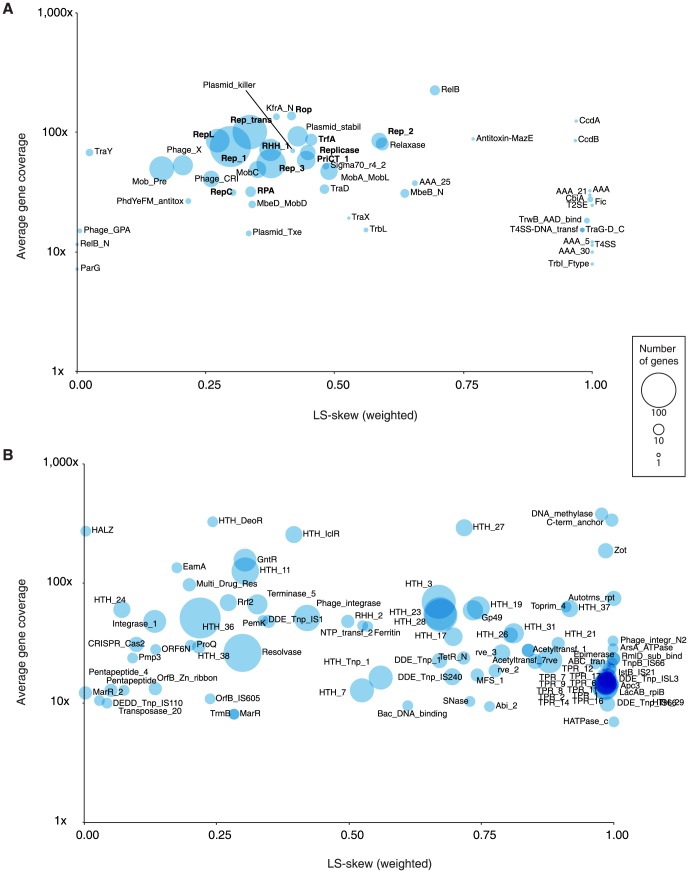
Bubble diagram showing occurrences of known protein-family domains (Pfams) associated with either plasmid-selfish (a) or non-plasmid-selfish (b) functions among 1,119 genes predicted from a de novo assembly made from two wastewater metamobilomes. The two samples were isolated with either an established method (S) or a modified method using electroelution (L; this study). Each bubble represents a cluster of Pfam-domain containing proteins and is positioned according to the average coverage of all genes encoding that protein domain against a weighted mean of LS-skew values (see methods) in which gene coverages were used as weights. For non-plasmid selfish function (b), only clusters with two or more genes were included.

Putative gene functions were inferred by scanning all putative protein sequences for presence of conserved domains (Pfams) [29]. The number of protein sequences that contained at least one Pfam domain was 1,119, thus 69% of sequences contained no conserved domains. A total of 1,513 conserved domains from 323 different Pfams were found; these we divided into 50 plasmid-selfish and 273 non-plasmid-selfish Pfams (Table S3). Each individual Pfam with domain hits among the putative proteins was assigned weighted LS-skew values according to a weighting scheme factoring in gene coverages of genes encoding that particular protein domain (Figure 3). Based on the number of genes containing Pfam-domains associated with replication of circular elements (see discussion), and the assumption that the majority of plasmids contain only a single replication gene, it was estimated that the assembly contained at least 448 different plasmid replicons (295/127/26). Thus, by dividing the sums of contig lengths in each of the three fractions of the mixed assembly with the estimated number of replicons (S/both/L), the largest possible average plasmid sizes were estimated to be 10.5, 22.0 and 38.5 Kbp, respectively.

## Discussion

Metagenomic studies are setting the standard for non-culture-based characterizations of microbial communities, and recent years have seen increased interest in using this approach to characterize the fraction comprised of plasmids within such communities [9],[12]–[14],[19],[30]. However, good metamobilomes have proven elusive, as they are difficult to isolate without a contaminating presence of host chromosomal DNA (gDNA). Two recently published methods have shown that a relatively simple three-step procedure can serve as an effective means for procuring metamobilomes free of gDNA for metagenomic studies [12], [19]. However, the inclusion of an MDA step, which helps ensure sufficient DNA for high-throughput sequencing, provides a source of bias that could lead to an unfortunate overrepresentation of small plasmids. As a consequence, few of the studies that use MDA have reported recovery of sequences larger than 10 Kbp.

To demonstrate an overall robustness to their method, Kav *et al*. conducted an experiment which showed very similar enrichment of 3 model plasmids of different sizes (3, 8 and 65 Kbp, respectively) [17]. However, they did not address the effects of performing the same overall procedure on heterogeneous mixtures of plasmids, which would be more analogous to the intended target material (*i.e.* mobilomes). On their own, plasmids will be amplified similarly if MDA reactions are run until deoxynucleoside triphosphates (dNTPs) are depleted, and the same amount of DNA template is used to begin with. However, in the presence of multiple circular DNA templates, the Φ29 DNA polymerase, which utilizes a rolling-circle replication mechanism, will complete more trips around smaller circular templates than their larger counterparts. The processivity of the Φ29 DNA polymerase is such, that up to 70 Kbp products can be generated from a contiguous (circular) template before dissociation [31]. Theoretically, 20 copies of a 3.5 Kbp plasmid would therefore be generated for each copy of a 70 Kbp plasmid under optimal circumstances. Furthermore, at equal DNA concentrations, the solution would contain orders of magnitude more copies of the smaller plasmid to begin with.

To demonstrate how this might affect the composition of heterogeneous plasmid mixtures, we used two model plasmids, the 4.4 Kbp cloning vector pBR322, representing small plasmids in the range 1–10 Kbp, and the 56 Kbp IncP1-family plasmid pKJK10, representing large plasmids over 10 Kbp, to measure respective enrichment in separate isolations and in a mixture of the two plasmids. In agreement with Kav *et al.* [17], comparable DNA yields were observed when the procedure was performed on separate plasmids, but, rather crucially, the smaller plasmid displayed a 34-fold higher amplification rate than the larger plasmid when the overall procedure was applied to a corresponding plasmid mixture. Consequently, the mixed sample went from a 1∶1 composition to 97∶3 in favor of pBR322 when following the previously established isolation procedure. Each exonuclease reaction showed a relatively similar degree (62–80%) of DNA-removal to other samples, and similar DNA yields (200–500 ng product from 0.2 ng template) following MDA. The substantial lowering of pKJK10 recovery in the plasmid mixture is therefore most likely directly attributable to much greater amplification of pBR322 by the Φ29 DNA polymerase.

To compensate for the observed bias, we propose the introduction of a simple electroelution step to the isolation protocol, to be carried out immediately prior to exonuclease digestion. Briefly, purified pDNA from bulk plasmid purifications is separated using gel electrophoresis, and gel slices containing one or more desired size ranges of pDNA are cut from the gel, and subjected to electroelution. The step is designed to recover larger plasmids from the sample and shield them from competition from smaller plasmids during the subsequent MDA step. This was demonstrated on the same two plasmids as before, showing acceptable recovery (12%) of the larger plasmid, with a severely reduced presence of the smaller plasmid (0.07%). Thus, the suggested addition to the protocol did not eliminate the smaller plasmid from the sample, as a tiny fraction of these most likely co-migrated with larger plasmids and lingered within the upper part of the gel. However, we believe that the electrophoresis step, which was still able to ensure over 99% purity of pKJK10, could be optimized to improve this with relatively few modifications, such as reducing the concentration of agarose and running gels at a lower voltage. Alternatively, samples could be subjected to the more accurate, but also considerably more elaborate pulsed-field gel-electropohoresis (PFGE), in order to ensure maximum separation of differently sized plasmids.

The question remains to what degree the currently used isolation method, with its intrinsic small plasmid bias, affects downstream metagenomic data output, and how well the suggested modification to the protocol aids in the recovery of large, naturally occurring plasmids. We therefore constructed metamobilome libraries from a bacterial wastewater community, and sequenced them, using Illumina HiSeq technology, in order to gauge the data output of the suggested modified protocol (L) against the standard metamobilome (S) protocol. In addition to the L- and S-library, we constructed two similar libraries (Ss & Ls) from wastewater spiked with a 1∶1 mixture of stationary phase *E. coli* cells, which harbored either of the two model plasmids. This was done to evaluate the respective *in silica* recovery from highly heterogeneous plasmid mixtures.

Read recruitment to known plasmid sequences revealed the spiked libraries to consist predominantly of reads originating from pBR322 and pKJK10. The distribution between the two plasmids had shifted even more in favor of pBR322 in the Ss-library, as nearly 1,300 times as many reads mapped to pBR322 than pKJK10. This was also seen in the Ls-library, albeit with only ∼100 times as many pBR322 reads due to much improved recovery of pKJK10. Thus, despite the demonstrated virtual removal of the smaller plasmid with the addition of the electroelution step, the remaining minute amounts of small plasmid was still sufficient to significantly outcompete the large model plasmid in the subsequent MDA step. Crucially, however, despite displaying a strong presence of the small plasmid, the addition of the electroelution step led to complete mapping of the pKJK10 genome, to a depth of over 50×. Conversely, pKJK10 was too poorly mapped by the Ss-library to be considered recovered. The smaller plasmid pBR322, which derives from the ColE1-type pMB1 replicon, has been reported to maintain natural copy numbers of 15–20, while the IncP1-family RK2 replicon, which pKJK10 is closely related to, has been reported to maintain copy numbers of 4–7 in *E. coli* [32]–[34]. Consequently, pKJK10 in its lowest copy number would take up 2.5 as many nucleotides (224 Kbp) as pBR322 in its highest copy number (87 Kbp) per cell, despite existing in 5 times fewer copies. Furthermore, there is evidence suggesting that the pKJK5-derived pKJK10 suffers very little plasmid loss during exponential growth, even in the absence of antibiotic selection, by maintaining high rates of conjugation [35], [36]. Hence, we do not believe that differences in natural copy numbers or plasmid loss during overnight growth are likely major contributors to the considerable worsening of small plasmid bias observed here. Rather, it would seem, that introducing a large plasmid to a heterogeneous plasmid mixture containing myriad smaller plasmids shifts the odds even further against its favor when subjected to MDA. This underpins the need for good separation of differently sized plasmids during the initial gel electrophoresis, which we believe can with remedied using one or more of the suggested optimizations briefly mentioned above.

As we were also interested in evaluating the recovery of naturally occurring plasmids from the modified protocol, we calculated the proportional contributions of reads (LS-skew values) from each of the two non-spiked libraries that mapped to known plasmid sequences. Median plasmid sizes of partially- to completely covered plasmid genomes were estimated to be 7 times larger in the L-library than in the S-library (Table 3), consistent with the earlier observations that the electroelution step was required for complete recovery of pKJK10. Thus, there is a similar clear indication from the sequencing data, that the modified method much improves, in fact permits, the recovery of large plasmids. However, as nearly half of the plasmids with partial- to full coverage had significant mapping of reads from both libraries, there is also evidence suggesting a degree of overlap between the size ranges of the two methods. This is most likely a consequence of co-migrating small plasmids during the electroelution step, which, as observed above, are then subject to the observed strong amplification bias during MDA.

It should be noted that smaller plasmids were found, in general, to have noticeably better genome completeness (S-library median: 53%; L-library median 43%), than larger ones (S-library median: 30%; L-library median: 16%). Hence, out of the 61 RefSeq plasmids with near-complete to complete mapping, only 7 exceeded 5 Kbp in length, and only two exceeded 10 Kbp. The overall fragmented nature of large plasmid coverage is most likely a consequence of the relatively limited number of plasmid genomes present in the RefSeq database. Larger plasmids are often mosaic in nature, but will contain conserved regions concerned with plasmid-selfish functions [1], and are therefore more likely to have partial, or chimeric homologues in the RefSeq database. Interestingly, three of the smaller plasmid sequences with near-complete coverage were previously isolated from the human gut flora using transposon-aided plasmid capture (TRACA). This method has also been suggested as a viable option for characterizing metamobilomes [14], [30], [37], but has so far been very limited in its ability to capture upper size range plasmids.

Surprisingly, and also rather unfortunately, almost a quarter of the reads in the L-library mapped to the two model plasmids. This included a variant of the *A. victoria gfp*-gene (*gfp*mut3*) that is extremely unlikely to occur naturally on bacterial plasmids, which is a strong indication of contamination of the L-library with pKJK10. Furthermore, the sparse mapping of pBR322 and the complete absence of reads mapping to the *gfp*-gene in the S-library, suggests that the high occurrence of pBR322 reads in the L-library was not due to similar, but naturally occurring plasmids of the same type (*i.e.* ColE1- or IncP1-family). The L-library had therefore most likely been compromised by both model plasmids. This, we attribute to cross-contamination stemming from the highly spiked wastewater sample being run on the same gel as the non-spiked sample during the gel electrophoresis step. This most likely led the two model plasmids, which were highly abundant in the spiked sample, to seep into adjacent areas of the gel that was later excised in preparation for the L-library. This was confirmed by testing for the presence of model plasmids in adjacent lanes on a gel (following electroelution) where only the spiking mixture was run in a single lane. Furthermore, it appeared that the electroelution itself was also vulnerable to cross-contamination, as it was possible to test positive for pBR322 (and slightly pKJK10) by running the electroelution procedure on an empty gel slice, excised from an agarose gel run in a separate electrophoresis chamber, in the same electrophoresis chamber as gel slices excised from the previous pDNA positive gel. We therefore advise caution when running multiple samples on the same gel and recommend that samples be run on separate gels or at the very least have ample lane spacing on the gel, and finally are electroeluted separately.

This regrettable occurrence aside, the two libraries showed very similar proportions of reads mapping to known RefSeq plasmid sequences (4% and 6%, respectively), when not considering reads mapping to either of the two model plasmids.

As approximately 90% of the two non-spiked libraries (the proportion of unknown sequences in the L-library rose from 68% to 89% when not considering model plasmid reads) consisted of reads that did not map to any of the plasmid-, genome- or phage sequences present in the RefSeq library, an evaluation of *de novo* assembled metamobilome data was also performed. Due to the shortness of the sequencing reads (around 77 bp) and the choice of using single-read libraries, the assembled contigs were of limited length, although with a few slightly longer contigs assembled by reads coming from the L-library. It was however evident that the S-library produced a larger and more diverse set of contigs than the L-library, consistent with the S-library containing many small plasmids, and the L-library containing fewer, larger plasmids (among others the contaminant pKJK10 mentioned above).

Both libraries showed a strong presence of genes encoding putative plasmid-selfish traits (Figure 3a); most notably, those responsible for maintaining plasmid copy numbers, which were present in high numbers, and with high coverage. In particular the replication initiator [38] domains Rep_1 (rolling-circle mechanism), Rep_3 (iteron-binding mechanism), Rep_trans (rolling-circle), RHH_1 (present in CopG-family transcriptional activators) and RepL (Firmicute plasmid replication) were each present on more than 50 different proteins. Other replication-associated domains, such as RepC, RPA and TrfA, which are closely linked to a number of broad-host range plasmids [39], [40], were also present, but in smaller numbers, as were the Replicase and Rep_2 domains. Additionally, both libraries encoded proteins containing domains related to plasmid mobilization, in particular MOB-type relaxases [5] and various plasmid stability functions such as toxin-antitoxin- (e.g. RelB, Plasmid_Txe, CcdAB) and plasmid segregation systems (CbiA a superfamily which includes the ParA nucleotide binding domain) [41]. Thus, despite a relatively large proportion of predicted proteins being unknown, there was a strong evidence to suggest that both metamobilomes contained myriad plasmids; in excess of 400 unique replicons. A number of proteins that were exclusively linked with the L-library, contained domains associated with mate-pair formation, such as type IV-secretion systems (AAA, T4SS, TraG-D) and T4SS-relaxosome coupling (TrwB_AAD_bind), which are notable features of conjugative plasmids [5]. Due to the number of genes involved in plasmid conjugation, the occurrence of such genes is strong evidence for the presence of large plasmids [42].

Non plasmid-essential genes showed a more dispersed distribution of encoded protein domains, indicating a distinct difference in gene content between the S- and L-libraries (Figure 3b). Overall, these genes were dominated by a presence of helix-turn-helix (HTH) motifs, suggesting involvement in gene regulation [43], and multiple transposases and integrases (DDE_Tnp, rve), suggesting presence of multiple transposable elements. Interestingly, most motifs associated with transposable elements and gene regulations were skewed towards the L-library, consistent with larger plasmids being able to accommodate large inserted regions such as transposons and integrons [1], as well as operons, which are normally under tight regulation by repressor- or activator genes.

## Conclusions

The current study points out a severe bias associated with using metamobilome/plasmidome isolation methods based on exonuclease digestion followed by MDA. As previously demonstrated, it can lead to underrepresentation of naturally occurring plasmids in the upper size range (>10 Kbp), when left unchecked. As a result, metagenomic data produced with this method are likely to be completely dominated by smaller cryptic or mobilizable plasmids [42] from the lowest size range (<10 Kbp), excluding a large and significant portion of the accessory gene content contained within microbial communal gene pools. Small plasmid data, while potentially useful in select cases (such as screening for new potential cloning vectors or resistance genes), would be less relevant to studies concerned with lateral gene transfer, local environment adaptions and genome evolution. The addition of an electroelution step to the isolation protocol, in which different size ranges of plasmids are enriched separately, provides an update to a relatively simple and high-throughput protocol. As shown by this study, the resulting data output adds an important look into the upper size range of naturally occurring plasmids. Our data show that in addition to providing insight into plasmid-selfish functions associated with larger conjugative plasmids, it also gives access to a large and potentially important pool of hitherto uncharacterized accessory genes.

## Supporting Information

Table S1 **Results from mapping of metamobilome sequencing reads using the Burrows-Wheeler Alignment program (BWA).**
(XLSX)

Table S2 **Summary of read recruitment of the metamobilome libraries S, L, Ss and Ls to the NCBI RefSeq database of completed plasmid sequences.**
(XLSX)

Table S3 **Coverages and LS-skew values (see methods) of genes encoding Pfam-domains associated with plasmid-selfish or non-plasmid-selfish functions.** Weigthed LS-skew values factor in the gene coverages of all genes assigned to a given Pfam cluster.
(XLSX)
